# Adaptive Symmetry Self-Matching for 3D Point Cloud Completion of Occluded Tomato Fruits in Complex Canopy Environments

**DOI:** 10.3390/plants14132080

**Published:** 2025-07-07

**Authors:** Wenqin Wang, Chengda Lin, Haiyu Shui, Ke Zhang, Ruifang Zhai

**Affiliations:** 1 College of Resources and Environment, Huazhong Agricultural University, Wuhan 430070, China; wenqin@webmail.hzau.edu.cn (W.W.); shuihaiyu@webmail.hzau.edu.cn (H.S.); zk1976023128@126.com (K.Z.); 2 College of Informatics, Huazhong Agricultural University, Wuhan 430070, China

**Keywords:** adaptive symmetry self-matching, point cloud completion, symmetry analysis, tomato phenotyping, smart agriculture

## Abstract

As a globally important cash crop, the optimization of tomato yield and quality is strategically significant for food security and sustainable agricultural development. In order to address the problem of missing point cloud data on fruits in a facility agriculture environment due to complex canopy structure, leaf shading and limited collection viewpoints, the traditional geometric fitting method makes it difficult to restore the real morphology of fruits due to the dependence on data integrity. This study proposes an adaptive symmetry self-matching (ASSM) algorithm. It dynamically adjusts symmetry planes by detecting defect region characteristics in real time, implements point cloud completion under multi-symmetry constraints and constructs a triple-orthogonal symmetry plane system to adapt to multi-directional heterogeneous structures under complex occlusion. Experiments conducted on 150 tomato fruits with 5–70% occlusion rates demonstrate that ASSM achieved coefficient of determination (R^2^) values of 0.9914 (length), 0.9880 (width) and 0.9349 (height) under high occlusion, reducing the root mean square error (RMSE) by 23.51–56.10% compared with traditional ellipsoid fitting. Further validation on eggplant fruits confirmed the cross-crop adaptability of the method. The proposed ASSM method overcomes conventional techniques’ data integrity dependency, providing high-precision three-dimensional (3D) data for monitoring plant growth and enabling accurate phenotyping in smart agricultural systems.

## 1. Introduction

Driven by the strategic needs of global food security and sustainable agricultural development, the precise acquisition of crop phenotypic information is gradually transforming from macro observational statistics to micro three-dimensional (3D) reconstruction and intelligent phenotyping. The Food and Agriculture Organization of the United Nations predicts that global food demand will increase by about 60% by 2050, while arable land and freshwater resources are under constant pressure, forcing agricultural production to accelerate the transition to digitalization, precision and efficiency [1]. As a direct external representation of crop growth status, the accurate judgement of phenotypic parameters is not only related to yield assessment and quality grading but also an important basis for the optimal allocation of agricultural resources, intelligent decision making and carbon reduction [2,3,4]. As an important cash crop widely grown globally with high nutritional value and abundant production, tomato has long played a key role in human nutritional security and regional agricultural economic development [5,6,7]. However, a key challenge in plant phenotyping is the integrity of 3D data for fruit under complex canopy conditions. Recent studies have shown that occlusion from leaves and stems can cause a point cloud loss of up to 60%, which substantially reduces the accuracy of phenotypic measurements such as fruit volume and surface area. Especially when monitoring the dynamics of fruit or stress-responsive phenotypes during the expansion stage, missing data may mask the signals of key physiological changes [8,9,10].

Current fruit point cloud completion methods mainly include the geometric fitting method, symmetry-based completion method and deep learning-based 3D reconstruction method. The geometric fitting method reconstructs the fruit morphology by fitting spheres, ellipsoids or other regular geometric shapes, and it is highly sensitive to missing points, although it achieves high reconstruction accuracy with complete point cloud data. Its parameter estimation error increases substantially as the degree of occlusion increases, and it is poorly adapted to the morphology of deformed or asymmetric fruits [11,12,13,14]. Symmetry-based methods utilize the morphological symmetry of fruits to recover missing regions by mirroring across a fixed-axis or single-symmetry plane. This strategy improves completion to a certain extent [15,16,17,18]. However, these methods generally assumed that fruit symmetry remains constant during growth, ignoring the dynamic changes in morphology during the expansion period or under environmental stress. As a result, pose estimation may become biased, and the error is further amplified in high missing rate scenarios. The single-symmetry face completion method only relies on a single-symmetry dimension, which is difficult to deal with the complex situation of simultaneous occlusion of the top leaves and lateral stalks, leading to a significant increase in the root mean square error (RMSE) of the lateral reconstruction and accompanied by the problem of topological structure breakage or excessive smoothing [18]. In recent years, some scholars have attempted to introduce deep learning-based point cloud reconstruction methods, such as point cloud completion techniques based on PointNet, F-PointNet, or NeRF frameworks for deep learning. These methods have shown potential for fruit reconstruction in open environments, but their generalization ability in complex multi-obscuration scenes remains limited and relies on large-scale training data support [19,20,21,22,23].

In summary, the existing fruit point cloud completion methods lack a multi-dimensional symmetry–dynamic optimization mechanism when coping with multi-directional heterogeneous defects in fruit point clouds under complex canopy environments and rely on static single-symmetry assumptions. This limitation compromises their adaptability to multi-directional defective scenarios. Crucially, a multi-orthogonal symmetry–dynamic completion system has not been established, and the adaptability and accuracy of complementary local missing regions require improvement. To address these challenges, there is an urgent need to develop a point cloud completion method that integrates the inherent symmetry characteristics of fruit morphology, the multi-orthogonal symmetry plane dynamic completion mechanism, and the adaptability of heterogeneous defective structures. This approach aims to improve the accuracy and application value of the 3D morphological completion of fruits and the efficient analysis of phenotypic information in a facility-based agricultural environment.

Aiming at solving the above challenges, this study proposes that the adaptive symmetry self-matching (ASSM) algorithm, which detects the symmetry distribution characteristics of defective regions in real time, dynamically adjusts the number, position and orientation of symmetry planes and constructs a multi-symmetry plane cooperative completion system. This framework achieves personalized completion of missing regions in complex occlusion environments and accurately restores the true morphology of occluded fruits. Tomato was selected as the representative fruit in this study, a two-layer architecture of “local symmetry detection-global morphological optimization” was established, and the method’s ability to complete fruits in a point cloud with a high missing rate was validated. The method was further migrated to eggplants to explore the cross-species adaptability. This work introduces, for the first time, a multi-symmetry adaptive dynamic matching mechanism into the 3D phenotyping analysis of plants. It not only solves the point cloud completion problem under an occlusion scenario but also supports the refinement research, such as the classification of fruit developmental stages and the detection of morphological aberrations, which can provide a high-confidence database for plant physiologists. The main contributions of this study are as follows: (1) proposal of the ASSM algorithm, which introduces the multi-symmetry adaptive dynamic matching strategy for the first time, breaking through the limitation of single-symmetry surface static completion; (2) constructs a multi-symmetry surface collaborative completion system, which significantly improves the validity and accuracy of the completion in complex occlusion environments; (3) implements a cross-species validation system of the method and migrates and applies this symmetry completion method to an eggplant fruit point cloud to investigate its adaptability and completion accuracy in different crops.

## 2. Materials and Methods

### 2.1. Experimental Setup and Sample Acquisition

**Figure 1:**
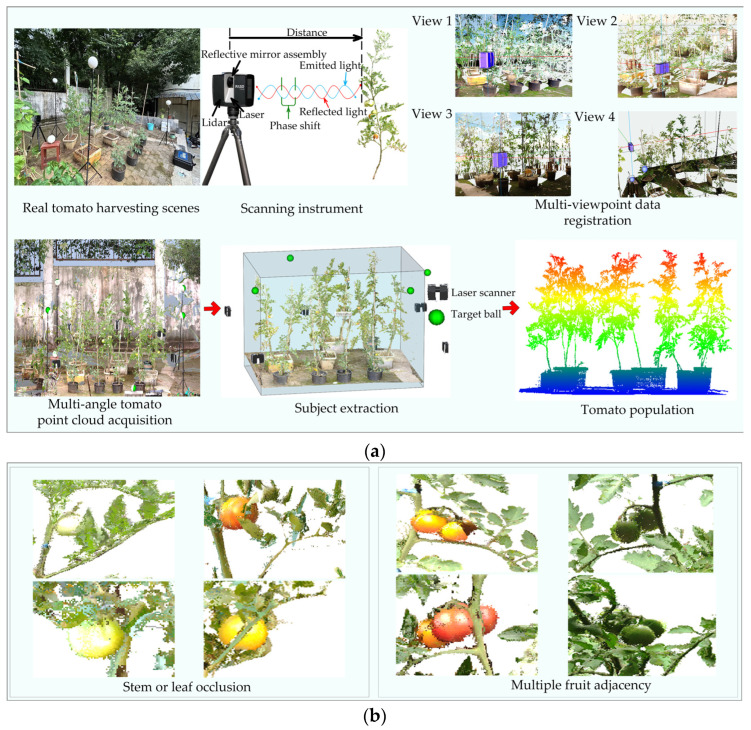
3D laser scanning setup for tomato plant point cloud acquisition: (**a**) tomato population acquirement; (**b**) diagram of dense stem and leaf coverage.

This experiment was conducted from April to August 2024 at the College of Resources and Environment, Huazhong Agricultural University, Hongshan District, Hubei Province, China. The tomato cultivar used in this study was “Xuanli F_1_”, a widely cultivated red-fruited hybrid developed by Tiandun Seed Company (Jinan, China). It is an early- to mid-maturing variety with an indeterminate growth habit and produces uniformly sized fruits with a low incidence of malformation. It is one of the most commonly grown red-fruited tomato hybrids in China. A FARO Focus S70 (FARO, Lake Mary, FL, USA) 3D laser scanner was used to collect point cloud data of mature tomato plants. The operational parameters of the scanner are provided in Table 1. All scans were conducted outdoors under natural daylight conditions without artificial lighting during the 15th to 21st week of tomato growth, at which stage the plant heights ranged from 1.2 m to 2.0 m. The study area was scanned using multiple light detection and ranging (LiDAR) stations to ensure that realistic scenes such as leaf shading and stem crossing were captured [24,25,26], as shown in Figure 1a, and the tomato population plant collection information is shown in Table 2. As shown in Figure 1b, due to the dense plant canopy, some fruit point clouds exhibited varying degrees of occlusion.

To ensure the statistical adequacy of the sample size for detecting meaningful differences in fruit morphology completion accuracy across different occlusion levels, an a priori power analysis was conducted using G *Power 3.1 software. An effect size of *f* = 0.35 was specified based on prior studies on plant phenotyping and 3D point cloud completion under occluded conditions [27,28], where medium to large effect sizes were typically observed. This effect size reflects a moderate to substantial difference in the mean completion errors relative to the within-group variability across different occlusion scenarios. With a significance level of *α* = 0.05 and a desired statistical power of 0.95, the analysis indicated a minimum required sample size of 138 fruits. Therefore, the inclusion of 150 fruit samples in this study was deemed statistically sufficient to ensure the robustness and generalizability of the experimental findings. Accordingly, 150 tomato fruit samples were included in this study, which met the statistical requirement for robust analysis.

### 2.2. Fruit Occlusion Rate Calculation and Classification

To objectively assess the impact of occlusion on 3D fruit completion, the occlusion rate for each sample was first calculated. The occlusion rate was defined as the proportion of missing surface area relative to the estimated complete surface area of the fruit and computed using the following formula:

(1) $$O\mathrm{cclusion}\;\mathrm{Rate}\;=1-\frac{A_{observed}}{A_{complete}},$$

where *A_observed_* represents the surface area obtained from the partially occluded point clouds, and *A_complete_* represents the estimated surface area of the corresponding complete fruit.

To compute *A_complete_*, 20–30 multi-view RGB images of each fruit were captured from different angles and processed using structure-from-motion and multi-view stereo techniques to reconstruct dense 3D point clouds. Poisson surface reconstruction was then applied to generate a triangular mesh, and the total surface area was calculated using the Trimesh Python library (version 3.21.5) under Python 3.10 [29].

The same surface reconstruction method was applied to the occluded point clouds to ensure consistency in geometry estimation when calculating *A_observed_*_._

In this study, the occlusion rate of the samples ranged from 5% to 70%. Based on the distribution of occlusion rates, samples were classified into three categories, as shown in Table 3. Although no universally accepted standard exists for occlusion classification, the thresholds used in this study were empirically determined based on the distribution of occlusion conditions in the dataset to ensure balanced and representative grouping. Similar occlusion-based grouping strategies have been adopted in recent 3D plant reconstruction studies [30], which support the methodological validity of this approach.

### 2.3. Point Cloud Preprocessing

3D laser scanning technology offers high accuracy, high resolution, and wide-range scanning capability; however, the acquired point cloud data often contain environmental noise and non-target vegetation [31]. In this study, statistical filtering was applied to eliminate sensor noise, and straight-through filtering was used to remove background interference, thereby enabling accurate plant segmentation [32,33]. To improve the processing efficiency, the point cloud density was reduced by voxel downsampling while preserving key morphological features.

### 2.4. Tomato Fruit Extraction

**Figure 2:**
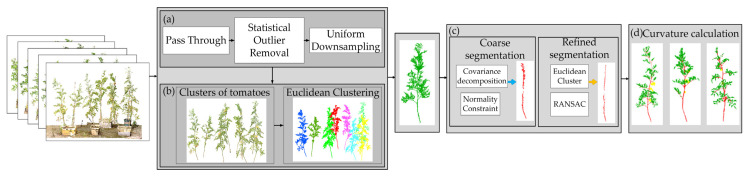
Tomato fruit extraction: (**a**) point cloud preprocessing; (**b**) single-plant segmentation; (**c**) stem extraction; (**d**) fruit extraction.

In this study, the Euclidean clustering algorithm was employed to segment individual tomato plants by grouping spatially adjacent points based on Euclidean distance [34]. Considering the vertical growth characteristics of the stalks, a local neighborhood covariance matrix was constructed, and eigenvalue decomposition was performed. Points deviating from the main stem were preliminarily excluded by applying normal vector-based constraints [35]. The stem geometry was further refined using Euclidean clustering combined with cylindrical model fitting to achieve high-precision extraction (Figure 2c). Fruit extraction was based on curvature features. The point cloud normals were first calculated, and the local curvature was estimated using principal component analysis (PCA). A region-growing algorithm was then applied to expand from high-curvature seed points, with the curvature and smoothness thresholds dynamically adjusted to accommodate different fruit sizes [36], as shown in Figure 2d.

### 2.5. Adaptive Symmetry–Dynamic Matching

**Figure 3:**
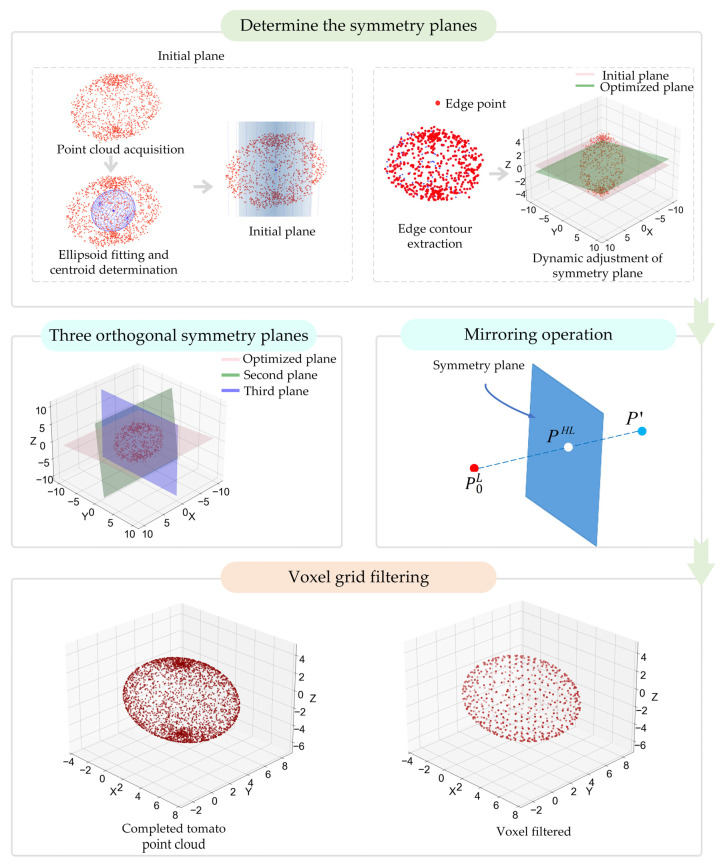
Adaptive symmetry self-matching algorithm for point cloud completion.

In this study, we propose a dual-architecture framework of “local detection-global morphological optimization” and introduced the ASSM algorithm to accurately locate the fruit symmetry plane using a combination of global constraints and local feature optimization strategies. The ellipsoid model was fitted to the tomato point cloud using the random sample consensus (RANSAC) algorithm, which determined the geometric center of the fruit and served as a spatial reference for symmetry plane estimation. PCA was then performed to calculate the point cloud covariance matrix, and the eigenvector corresponding to the maximum variance was extracted as the normal vector of the initial symmetry plane. Using this initial symmetry plane as a reference, one half of the point clouds was mirrored, and edge contours were extracted from both the unmirrored and mirrored point clouds using a K-dimensional tree (K-D tree) search. The iterative closest point (ICP) algorithm was subsequently applied to minimize the positional deviation between the two sets of contours, thereby dynamically optimizing the orientation of the symmetry plane. Once optimized, two additional orthogonal symmetry planes were generated, and the missing regions were mirrored from multiple directions under the constraints of the resulting tri-planar symmetry system. Finally, voxel-based mesh filtering was used to downsample and smooth the overlapping regions to reconstruct a complete tomato fruit model (Figure 3).

#### 2.5.1. Calculation of the Initial Symmetry

Tomato fruits are typically ellipsoidal and exhibit strong morphological symmetry [37,38,39]. Leveraging this property, an ellipsoid fitting technique was applied to obtain the geometric center of the tomato fruits. Ellipsoid fitting was performed using the RANSAC algorithm, which was iteratively optimized to minimize the fitting error between the point cloud and the ellipsoid surface. The geometric center of the tomato fruit was then determined.

After determining the spherical center of the tomato fruit, PCA was used in this study to initially determine the symmetry plane of the tomato fruit. The point cloud data were centered, the covariance matrix was calculated, and eigenvalue decomposition was performed. The eigenvector in the direction of the maximum variance was extracted as the symmetry plane normal vector [40,41]. Due to the inherent symmetry of tomato morphology, this principal eigenvector typically aligns with the natural symmetry axis of the fruit [42,43], forming the basis for the initial symmetry plane.

#### 2.5.2. Adaptive Dynamic Matching Optimization

**Figure 4:**
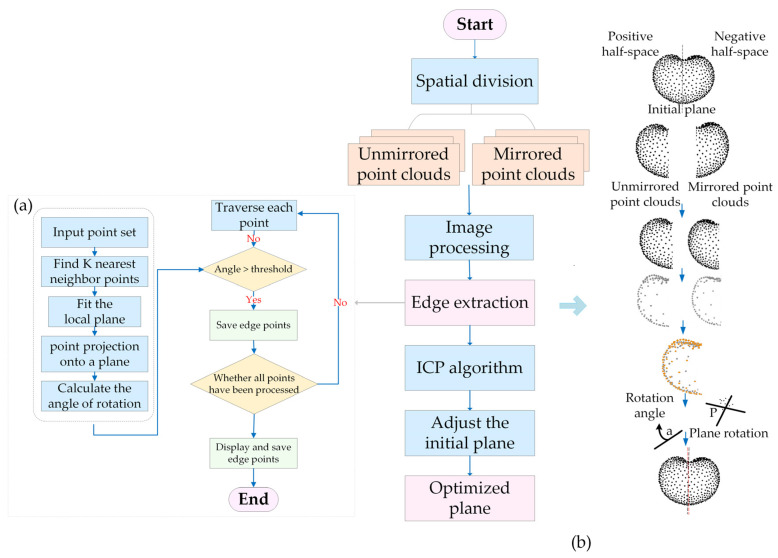
ASSM dynamic matching optimization process: (**a**) contour point extraction process; (**b**) initial plane optimization.

To further optimize the symmetry plane, the adaptive symmetry–dynamic matching algorithm was introduced. The full workflow is illustrated in Figure 4 and includes spatial division, edge contour extraction, iterative alignment, and symmetry plane adjustment. Initially, the fruit point cloud was divided into two spatial regions based on the normal vector of the preliminarily estimated symmetry plane. For point *P_i_ = (x_i_, y_i_, z_i_)* in the point cloud, its projection with respect to the plane of symmetry can be expressed as follows:

(2) $$D_{i}=(x_{i}-C_{x})n_{x}+(y_{i}-C_{y})n_{y}+(z_{i}-C_{z})n_{z},$$

where *D_i_* is the projection of the point *P_i_* onto the normal vector of the symmetry plane, and (*n_x_, n_y_, n_z_*) is the normal vector of the initial symmetry plane. When *D_i_* > 0, *P_i_* lies in the positive half-space; when *D_i_* < 0, *P_i_* lies in the negative half-space (Figure 4b, top).

Based on the above method, the total number of point clouds on each side of the symmetry plane was then calculated. Assuming that the point cloud dataset contains *N* points, the number of point clouds located in the positive half-space of the normal vector of the symmetry plane was calculated to be *N^+^*, and the number of point clouds located in the negative half-space was *N^−^*.

(3) $$\left\{\begin{array}{l} N^{+}=\sum_{i=1}^{N}I(Di>0)\\ N^{-}=\sum_{i=1}^{N}I(Di<0) \end{array}\right.,$$

where *I* is the indicator function that returns 1 when *D_i_* > 0 and 0 otherwise, and 1 for *D_i_* < 0 and 0 otherwise.

The side of the symmetry plane containing more points was selected for mirroring. Edge contours were extracted from both the original and mirrored point clouds using the K-D tree nearest neighbor algorithm [44]. The unmirrored edge points were defined as the unmirrored contours, and the mirrored edge points as the mirrored contours. The contour point extraction process is shown in Figure 4a, based on the least squares fitting of the plane, calculating the projection of the point to the plane and the local rotation angle, and thresholding to filter the edge points.

After contour matching, the ICP algorithm was applied to align the mirrored and unprocessed contours. This process dynamically adjusted the alignment step size and tolerance thresholds in real time to prevent local minima and preserve alignment accuracy. The rotation matrix and translation vector were iteratively optimized to achieve accurate registration between the point clouds [45]. Following alignment, the initial symmetry plane was rotationally transformed based on the rotation matrix calculated by ICP to obtain the final optimized symmetry plane. The optimized symmetry plane was slightly different from the initial one (Figure 4b, bottom). This optimized plane served as the basis for constructing a triple-orthogonal symmetry system in the final completion process. The normal vector *N_f_* of the final optimized symmetry plane can be calculated as follows:

(4) $$N_{f}=R*N_{0},$$

where *R* is the rotation matrix computed by ICP alignment, and *N*_0_ is the normal vector of the initial symmetry plane.

### 2.6. Triple-Orthogonal Symmetry Plane Synergy and Mirror Completion

#### 2.6.1. Construction of Three Orthogonal Symmetry Planes

In practical tomato fruit completion tasks, although a single-symmetry plane can partially recover missing regions, relying solely on one plane may result in morphological deviations from the actual fruit structure due to the diverse occlusion patterns and localized point cloud gaps. To address this problem, we proposed a point cloud completion method based on three orthogonal symmetry planes, combined with the ASSM algorithm. The approach enhanced both the completeness and morphological authenticity of the completion by introducing multi-directional symmetry constraints. Using the optimized symmetry plane as a reference, two additional symmetry planes that were orthogonal to the first and intersected the fruit’s centroid were constructed, forming a multi-directional constraint system spanning the 3D space.

#### 2.6.2. Point Cloud Mirroring and Final Integration

After constructing the symmetry plane system, the missing regions were mirrored along the three planes to generate multi-directional complementary point clouds. Considering the slight deviation of the symmetry plane estimation, ICP alignment was performed to refine the position to ensure that the complementary region was seamlessly aligned with the original point cloud. After completion, the overlapping points were downsampled and smoothed by voxel grid filtering to eliminate the redundant points and retain the morphological features, resulting in a complete and continuous fruit point cloud.

## 3. Results

### 3.1. Effectiveness of Tomato Fruit Extraction

In the tomato fruit point cloud extraction experiments, by processing the point cloud data of 42 tomato plants, high-precision segmentation of a single plant based on the Euclidean clustering algorithm was achieved with an accuracy of 97.62%. For each single-plant point cloud, 95.24% of stem points were eliminated by combining local neighborhood covariance matrix eigen analysis with normal vector direction constraints, significantly reducing stem interference during fruit extraction. The fruit point clouds were further extracted using curvature feature analysis and a regional growth algorithm, achieving an extraction accuracy of 96.15%. Even in scenarios of missing data, the method can still extract the tomato fruit point cloud stably, which lays a reliable data foundation for the subsequent morphological analysis and completion.

### 3.2. Symmetry Plane Verification

**Figure 5:**
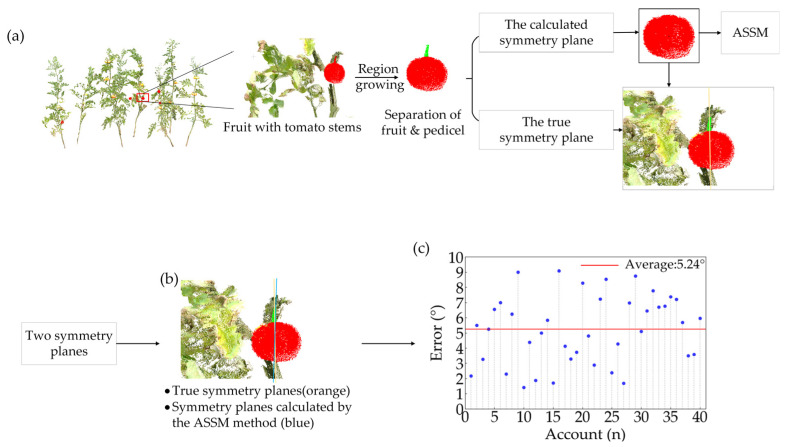
Verification of symmetry plane accuracy: (**a**) generation of calculated symmetry plane and true symmetry plane; (**b**) visual comparison between calculated symmetry plane and true symmetry plane; (**c**) symmetry plane angle error.

The tomato fruit peduncle is centrally located at the top of the fruit with a stable orientation, which is an important sign of the natural symmetry of the fruit [46,47]. Based on the morphological characteristics and growth patterns of tomato fruits, the position and orientation of the peduncle were used to calibrate the true symmetry plane of the fruit. In this study, 40 unobstructed fruits with intact peduncles were manually screened from the collected tomato fruit dataset. The direction of each peduncle axis was used as the true symmetry plane benchmark for the accuracy analysis of symmetry plane calculation. The ellipsoid fitting method was used to determine the geometric center of each fruit. The normal vector of the symmetry plane was calculated according to the direction of the axis of the tomato peduncle so as to determine the true symmetry plane of the fruits. The results show that the symmetry plane positioning method based on the ASSM algorithm, which iteratively adjusted the symmetry direction, enabled the calculated plane to accurately pass through the geometric center of the fruit and align with the peduncle direction (Figure 5b). The angular error between the calculated and true symmetry planes ranged from 1.41° to 9.08°, with an average of 5.24 ± 2.23°, which was lower than the 6.5° reported in a sweet pepper study. These results verify the effectiveness of the ASSM algorithm in symmetry feature matching and symmetry plane localization for tomato (Figure 5c).

### 3.3. Tomato Fruit Completion and Accuracy Analysis

**Figure 6:**
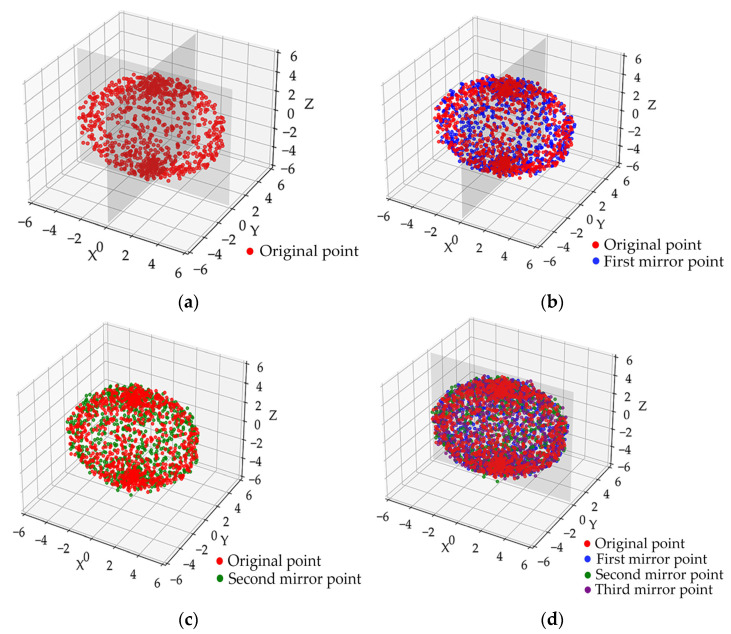
Multi-plane fruit completion integrated with ASSM: (**a**) fruit original point; (**b**) completion of edges and fusion of data using the first symmetry plane; (**c**) completion of top or bottom based on the second symmetry plane; (**d**) completion of lateral misses by the third symmetry plane.

In many studies, the morphological parameters of the fruit were usually calculated using ellipsoid fitting directly applied to the fruit surface. However, this fitting method is highly dependent on the completeness of the point cloud data. When parts of the point cloud were missing, the estimation of the ellipsoid semiaxis parameters became inaccurate, often leading to large deviations between the fitted and actual fruit morphologies. Therefore, direct ellipsoid fitting typically failed to produce accurate results. In this study, based on the symmetry of the fruit, the geometric center of the fruit was first obtained using ellipsoid fitting. PCA was then applied to determine the initial axis of symmetry, followed by ICP-based optimization to generate a three-orthogonal symmetry plane system. The missing regions were subsequently completed using mirror-based processing to reconstruct the tomato fruit, as illustrated in Figure 6.

In order to improve the alignment accuracy between the complementary effect and the boundary of the symmetric region, the ASSM algorithm was introduced. This method dynamically adjusted the location and extent of the mirror complementary region by iteratively calculating the Euclidean distance and density difference between the point clouds in the complementary region and the mirrored point clouds, effectively addressing boundary errors and symmetry shift problems existing in the traditional static mirror complementary.

**Figure 7:**
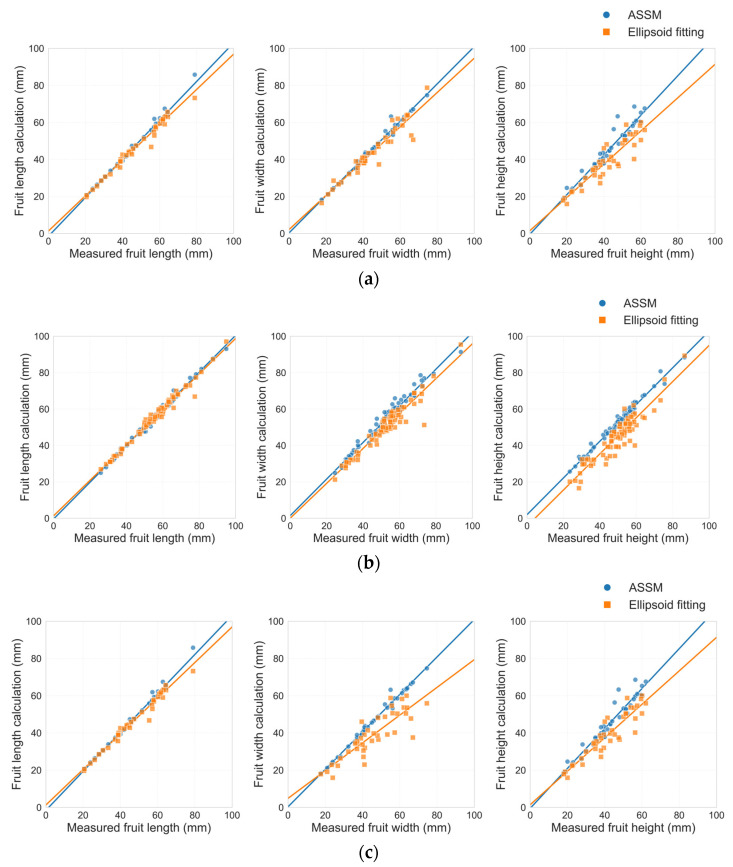
Comparison of calculated and measured values for tomato fruit size parameters under different occlusion levels: (**a**) low-occlusion level; (**b**) middle-occlusion level; (**c**) high-occlusion level.

As shown in Table 4, the coefficient of determination (R^2^) of all dimensional parameters for the 150 tomato fruits under low-, medium-, and high-occlusion levels was significantly higher using the ASSM method than with the ellipsoid fitting method. Under high occlusion, the R^2^ for fruit length and width approached 0.99. The R^2^ values of the ellipsoid fitting method under high occlusion were 0.9805, 0.9101, and 0.8489. The RMSE for all dimensions obtained using the ASSM method was lower than that of the ellipsoid fitting method, with the RMSE for fruit length under medium occlusion reduced to 1.1 mm. The relative root mean square error (rRMSE) values of the proposed completion method were also lower than those of the ellipsoid fitting method. To statistically evaluate whether these differences in prediction errors between the two methods were significant, the Wilcoxon signed-rank test was applied to the paired absolute errors of each fruit sample under the same occlusion level. The results show that for most dimensional parameters and occlusion levels, the differences were statistically significant (*p* < 0.05) or highly significant (*p* < 0.001). These findings confirm that the ASSM method achieved superior completion accuracy compared to the ellipsoid fitting method across varying occlusion conditions. To visually compare the prediction accuracy of the two methods under different occlusion levels, the calculated and measured values of the fruit size parameters were plotted. The ASSM method is more accurate under different occlusion levels, as demonstrated in Figure 7.

## 4. Discussion

### 4.1. Accuracy of the Method of Determining the Plane of Symmetry

In this study, we proposed a symmetry plane localization method that integrated global geometric constraints and local optimization. The initial symmetry plane was determined by ellipsoid fitting and PCA, followed by orientation refinement using ICP iterations with mirrored point cloud contours. Additionally, the ASSM algorithm was introduced to adaptively adjust the symmetry plane normal vector fine-tuning step size and the alignment weights based on missing point cloud data and contour differences so as to improve the robustness of the symmetry plane localization. The experimental results show that the average error between the symmetry plane determined by the method in this paper and the real symmetry plane is 5.24°, which outperformed the 6.5° error reported in the sweet pepper study. The ASSM algorithm effectively corrected symmetry plane offsets caused by locally missing point cloud by dynamically evaluating matching errors of the mirrored point cloud, significantly improved the accuracy of symmetry plane localization in 3D space. This method provides a stable and reliable basis for fruit completion and morphometry.

### 4.2. Comparison of Tomato Fruit Completion Methods

**Figure 8:**
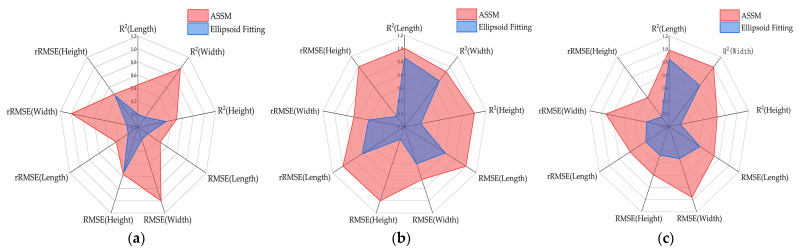
Radar chart comparison of the error in tomato fruit completion under different occlusion levels using two methods: (**a**) low-occlusion level; (**b**) middle-occlusion level; (**c**) high-occlusion level.

In this study, we compared two fruit completion methods based on ASSM and ellipsoid fitting. The results showed that the ASSM method was better than ellipsoid fitting in R^2^, RMSE and rRMSE metrics. Especially under high occlusion conditions, the ASSM algorithm dynamically optimizes the symmetry plane to ensure accurate alignment between mirrored and unmirrored contours, thereby improving completion accuracy. To statistically validate these observed differences in completion accuracy, the Wilcoxon signed-rank test was conducted on the paired absolute errors of the two methods for each fruit dimension under the same occlusion level. The statistical significance verified by the Wilcoxon signed-rank test further supported the conclusion that the ASSM method provided a more reliable and stable solution for fruit point cloud completion under complex occlusion scenarios. To visually compare the performance of the two methods across multiple indicators, radar diagrams were generated after applying min–max normalization to all metric values. For normalization, all metrics were standardized on a relative scale, where greater values consistently reflected superior completion results. As shown in Figure 8a, the ASSM fruit completion method yielded higher R^2^ values, better the ellipsoid fitting method, and the overall error control ability was stronger although the RMSE and rRMSE were smaller. As illustrated in Figure 8b, the ASSM method produced lower RMSE and rRMSE values than the ellipsoid fitting method, indicating reduced prediction error. In Figure 8c, the ASSM method again performed better in terms of the R^2^, RMSE, and rRMSE, with only minor differences in some individual metrics. Overall, the radar diagrams confirmed that the ASSM method maintained consistently superior or comparable performance.

**Figure 9:**
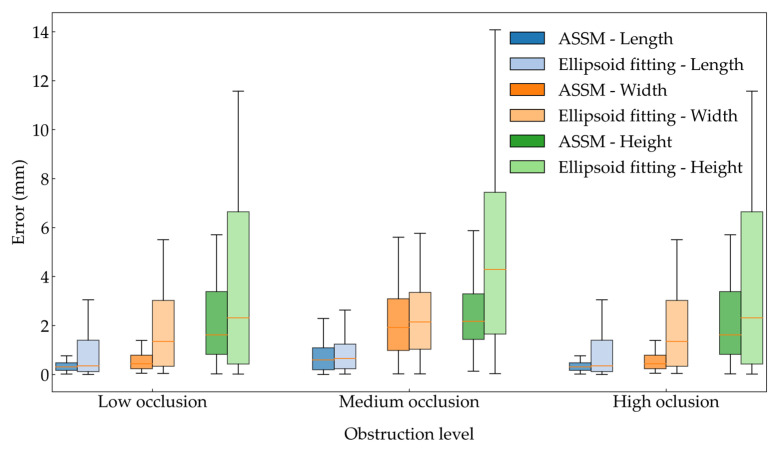
Box plot comparison of error rates for tomato fruit completion under different occlusion levels using two methods.

The box plots were generated to further compare the error distributions of the two methods, as shown in Figure 9. The results show that under all occlusion levels, the ASSM completion method produced lower box positions and shorter box lengths across the three dimensions. The error distribution was more concentrated with smaller values, particularly under high-occlusion conditions. Overall, the method demonstrates significantly greater stability and accuracy compared with the ellipsoid fitting approach.

These finding demonstrated that the ASSM method proposed in this paper more accurately recovered the 3D morphology of crops. This advantage mainly stemmed from the fact that the symmetry method effectively utilized the natural symmetry features of fruits through local mirror completion under the constraint of triple-orthogonal symmetry planes, overcoming the dependence of the ellipsoid fitting on data completeness. While ellipsoid fitting was susceptible to the interference caused by locally missing point clouds due to its global modeling nature, this often led to biased estimation of the semi-axis parameters. In contrast, the ASSM method was able to accurately restore the true fruit morphology under occlusion by dynamically optimizing the mirror plane and applying morphological filtering. In this paper, the fruit completion method showed multi-dimensional application value in plant phenotyping analysis. Through high-precision point cloud completion, it enabled dynamic monitoring of fruit development and assessment of abiotic stress impacts, while also providing 3D data for high-throughput phenotyping platforms supporting genotype–phenotype studies and precision breeding optimization.

### 4.3. Validation of Cross-Crop Applicability Based on Fruit Completion Methods

To further validate the effectiveness and generality of the ASSM completion method, this study applied it to the 3D completion of 30 eggplant fruits. The missing rates ranged from 10% to 70%, with an average of 54.10%. The ASSM algorithm was employed to adaptively adjust the symmetry plane optimization strategy, ensuring consistent completion performance across varying levels of missing data. As shown in Table 5, the R^2^ values for eggplant length, width, and height were close to 1, and both the RMSE and rRMSE values remained low. The ASSM algorithm enhanced completion stability under complex occlusion conditions through dynamic matching optimization. These results demonstrate that the method can be extended to apples, citrus and other fruits with symmetry features to achieve reliable 3D completion and phenotyping.

### 4.4. Comparison with Deep Learning-Based Completion Methods

While this study rigorously compared the performance of the proposed ASSM method against traditional ellipsoid fitting, deep learning-based point cloud completion approaches, such as PointNet++, PCN, and NeRF, were not included in the comparative analysis. This exclusion was primarily due to the substantial dependence of these models on large-scale annotated training datasets, their significant computational demands and the current lack of validation for densely occluded, high-density plant canopy environments. These limitations constrain their immediate applicability within controlled phenotyping pipelines. Moreover, the black-box nature of many deep learning models makes it challenging to interpret and explain the reconstructed geometry, particularly when analyzing subtle morphological traits required for phenotypic precision. In contrast, the ASSM method benefits from its explainable architecture and explicit use of geometric features such as symmetry planes, making it more transparent and easier to adapt for scientific analysis.

### 4.5. Limitations and Future Work

The proposed ASSM algorithm offers several distinct advantages. A primary strength was its ability to effectively address the challenges posed by high levels of occlusion by dynamically constructing and optimizing symmetry planes, leading to significantly improved completion accuracy over traditional ellipsoid fitting, as evidenced by R^2^, RMSE, and rRMSE metrics. Another advantage is its independence from large-scale annotated datasets, unlike deep learning-based approaches, which enhances its practicality for real-world applications. In addition, the method demonstrates strong cross-crop generalizability, as shown by its successful application to both tomatoes and eggplants, highlighting its adaptability to diverse fruit morphologies and complex occlusion scenarios.

A limitation of this work is that the diversity in fruit morphology may affect the accuracy of ASSM algorithms. In tomatoes of different varieties or grown under varying conditions, natural shape variations can obscure clear symmetry features. This, in turn, may reduce the accuracy of the symmetry plane estimation. In future work, we plan to improve the method’s robustness and adaptability to irregular or asymmetrical fruits by incorporating non-rigid registration techniques and deformable shape priors. Additionally, we aim to explore the integration of ASSM with deep learning models, such as incorporating symmetry constraints into neural network architectures like PointNet++ or PCN. This hybrid approach could leverage the interpretability of geometric methods alongside the generalization capability of data-driven techniques.

## 5. Conclusions

To address the problem of insufficient morphological restoration accuracy in traditional geometric fitting methods under fruit point cloud occlusion, this study proposes a 3D fruit completion method based on symmetry analysis. This method leverages the natural symmetry features of fruits and significantly improves the effect of point cloud completion under occluded environments. By integrating the PCA and the ICP algorithm, it achieved high-precision symmetry plane positioning with an average angular error of 5.24°. Based on this, a multi-orthogonal symmetry plane completion strategy was developed to generate mirrored point clouds for reconstructing the missing regions, thereby significantly enhancing the completion accuracy. The stability and cross-crop applicability of the method were demonstrated in 150 tomato fruits and eggplant crops and were better than the traditional ellipsoid fitting method. This study introduces the ASSM algorithm, which dynamically optimizes the alignment between the mirrored and unprocessed point clouds, thereby improving the consistency of symmetry plane positioning and the overall completion quality. The ASSM fruit completion method overcomes the limitations of traditional geometric models that rely heavily on complete data, thus enhancing the accuracy and reliability of 3D phenotypic analysis of fruits under complex occlusion conditions commonly encountered in field and greenhouse environments. This method holds significant promise for practical applications, including automated fruit grading, yield estimation, stress response monitoring, and 3D modeling of fruit growth dynamics in smart agriculture. Additionally, by enabling accurate extraction of multi-dimensional morphological parameters, it provides a robust foundation for genotype–phenotype association studies and precision breeding projects. Although the method performs well in the completion of symmetric fruit, it remains limited by its assumption of inherent morphological symmetry. This constraint may reduce its effectiveness when applied to fruits with irregular or asymmetrical shapes. In future work, based on the optimization of the ASSM algorithm, we will explore the completion strategies that incorporate local symmetry and shape priors, aiming to improve the accuracy and generalizability of the method for irregularly shaped crops.

## Figures and Tables

**Table 1:** On-site collection equipment’s operating parameters.

Category	Implementation Standard
Measurement range	0.6 m–70 m
Ranging error	±1 mm
Scanning speed	≤9.760 × 10^5^ dots/s
Scanning field of view	Horizontal 360° × vertical 300°
Operating temperature	20 ± 5 °C
Color scan	Average weighted metering

**Table 2:** Tomato population data information table.

Scan Date	Fruit Numbers	LiDAR Numbers	Plant Numbers
24 June 2024	10	5	8
15 July 2024	8	6	8
22 July 2024	8	5	10
28 July 2024	8	5	8
6 August 2024	8	4	8

**Table 3:** Distribution of occlusion levels in the tomato dataset.

Obstruction Level	Obstruction Ratio (%)	Fruit	Typical Occlusion Scenarios
Low	≤30	30	Partial leaf occlusion
Middle	<30–≤50	80	Top and side composite occlusion
High	<50–≤70	40	Dense multi-source occlusion

**Table 4:** Evaluation of the accuracy of two fruit completion methods.

Obstruction Level	Method	Dimensional Data	R^2^	RMSE (mm)	rRMSE	*p* *
Low	ASSM	Fruit length	0.9488	2.4000	0.0453	<0.001 ***
		Fruit width	0.9823	1.4810	0.0287	<0.001 ***
		Fruit height	0.9151	4.4514	0.0984	<0.001 ***
	Ellipsoid fitting	Fruit length	0.9124	2.8437	0.0536	0.009 *
		Fruit width	0.7784	5.7473	0.1114	0.047 *
		Fruit height	0.8895	4.5644	0.1009	<0.001 ***
Middle	ASSM	Fruit length	0.9933	1.1098	0.0199	0.1347
		Fruit width	0.9665	2.9495	0.0563	<0.001 ***
		Fruit height	0.9818	2.8425	0.0574	<0.001 ***
	Ellipsoid fitting	Fruit length	0.9816	1.8127	0.0326	0.3599
		Fruit width	0.9284	4.0436	0.0771	<0.001 ***
		Fruit height	0.8594	6.5552	0.1324	<0.001 ***
High	ASSM	Fruit length	0.9914	1.6261	0.0341	<0.001 ***
		Fruit width	0.9880	1.6442	0.0359	<0.001 ***
		Fruit height	0.9349	4.3583	0.1043	<0.001 ***
	Ellipsoid fitting	Fruit length	0.9805	2.1259	0.0446	0.009 *
		Fruit width	0.9101	4.3542	0.0951	0.047 *
		Fruit height	0.8489	5.5613	0.1331	<0.001 ***

* Indicates *p* < 0.05. *** Indicates *p* < 0.001.

**Table 5:** The completion effects of two fruit completion methods on eggplant.

Method	Dimensional Data	R^2^	RMSE (mm)	rRMSE
ASSM	Fruit length	0.9896	2.9072	0.0401
	Fruit width	0.9776	3.8759	0.0568
	Fruit height	0.9752	3.8095	0.0609
Ellipsoid fitting	Fruit length	0.9751	4.0022	0.0552
	Fruit width	0.9591	4.8265	0.0708
	Fruit height	0.9355	5.5238	0.0884

## Data Availability

The data are available online at https://doi.org/10.57760/sciencedb.25084.

## Abbreviations

The following abbreviations are used in this manuscript:

3D	Three-Dimensional
ASSM	Adaptive Symmetry Self-Matching
ICP	Iterative Closest Point
K-D tree	K-Dimensional Tree
LiDAR	Light Detection and Ranging
PCA	Principal Component Analysis
RANSAC	Random Sample Consensus
R^2^	Coefficient of Determination
RMSE	Root Mean Square Error
rRMSE	Relative Root Mean Square Error
